# Proteomics Investigation of the Time Course Responses of RAW264.7 Macrophages to Infections With the Wild-Type and Twin-Arginine Translocation Mutant Strains of *Brucella melitensis*

**DOI:** 10.3389/fcimb.2021.679571

**Published:** 2021-06-14

**Authors:** Xin Yan, Sen Hu, Yan Yang, Da Xu, Wenxing Liu, Ganwu Li, Wentong Cai, Zhigao Bu

**Affiliations:** ^1^ Key Laboratory of Veterinary Public Health of Ministry of Agriculture, State Key Laboratory of Veterinary Biotechnology, Harbin Veterinary Research Institute, Chinese Academy of Agricultural Sciences, Harbin, China; ^2^ Department of Veterinary Diagnostic and Production Animal Medicine, College of Veterinary Medicine, Iowa State University, Ames, IA, United States; ^3^ Jiangsu Co-innovation Center for Prevention and Control of Important Animal Infectious Disease and Zoonoses, Yangzhou, China

**Keywords:** *Brucella melitensis*, twin-arginine protein translocation, immune response, RAW264.7 cell model, cytokines, nitric oxide - NO

## Abstract

*Brucella*, a notorious intracellular pathogen, causes chronic infections in many mammals, including humans. The twin-arginine translocation (Tat) pathway transports folded proteins across the cytoplasmic membrane; protein substrates translocated by *Brucella* include ABC transporters, oxidoreductases, and cell envelope biosynthesis proteins. Previously, we showed that a Tat mutant of *Brucella melitensis* M28 exhibits reduced survival within murine macrophages. In this study, we compared the host responses elicited by wild-type M28 and its Tat-mutant strains ex vivo. We utilized label-free quantitative proteomics to assess proteomic changes in RAW264.7 macrophages after infection with M28 and its Tat mutants. A total of 6085 macrophage proteins were identified with high confidence, and 79, 50, and 99 proteins were differentially produced upon infection with the Tat mutant at 4, 24, and 48 hpi, respectively, relative to the wild-type infection. Gene ontology and KEGG enrichment analysis indicated that immune response-related proteins were enriched among the upregulated proteins. Compared to the wild-type M28 infection, the most upregulated proteins upon Tat-mutant infection included the cytosolic nucleic acid signaling pathway-related proteins IFIH1, DHX58, IFI202, IFI204, and ISG15 and the NF-*κ*B signaling pathway-related proteins PTGS2, CD40, and TRAF1, suggesting that the host increases the production of these proteins in response to Tat mutant infection. Upregulation of some proteins was further verified by a parallel reaction monitoring (PRM) assay. ELISA and qRT-PCR assays indicated that Tat mutant infection significantly induced proinflammatory cytokine (TNF-α and IL-6) and nitric oxide (NO) production. Finally, we showed that the Tat mutant displays higher sensitivity to nitrosative stress than the wild type and that treatment with the NO synthase inhibitor L-NMMA significantly increases the intracellular survival of the Tat mutant, indicating that NO production contributes to restricting Tat mutant survival within macrophages. Collectively, this work improves our understanding of host immune responses to Tat mutants and provides insights into the mechanisms underlying the attenuated virulence of Tat mutants.

## Introduction

*Brucella* are Gram-negative, facultative intracellular pathogens that can induce persistent infections in many mammals, such as livestock, wild animals, and humans. The disease caused by *Brucella*, brucellosis, manifests as reproductive disorders in animals, while in humans, this disease is characterized by a long incubation period that leads to a chronic, sometimes lifelong, debilitating infection with serious clinical manifestations, such as undulant fever, weakness and chronic inflammation of some organs, including the spleen and liver (Wang et al., 2011; Byndloss and Tsolis, 2016). Due to its high infectivity, *Brucella* has been classified as a potential agent of biological warfare, enhancing the growing interest in this pathogen’s biology, in particular as a model of complex intracellular parasitism (Robinson-Dunn, 2002).

A key aspect of *Brucella* pathogenesis is its interaction with macrophages (He et al., 2006; Byndloss and Tsolis, 2016). In many tissues, macrophages constitute the first line of defense of the innate immune response against invading microorganisms, including *Brucella* spp (Celli, 2006; Wang et al., 2017). Bactericidal responses of macrophages are activated by intracellular *Brucella*, including the fusion of phagosomes with lysosomes (Arenas et al., 2000) and the production of antimicrobial agents such as proinflammatory cytokines (TNF-α, IL-6, and IL-12), reactive oxygen intermediates (ROIs) and reactive nitrogen intermediates (RNIs) (Jiang and Baldwin, 1993; Jiang et al., 1993; Hop et al., 2017; Hop et al., 2019). In addition, in contrast to neutrophils and natural killer cells, macrophages were found to be the main cells involved in controlling brucellosis in a mouse model. In the early stage of mouse infection, macrophages allow the survival and replication of *Brucella*. With prolonged infection time, after the adaptive immune response is gradually established, macrophage antibacterial pathway components such as RNIs and ROIs are activated by IFN-γ and TNF-α, and activated macrophages then become the primary source of *Brucella* elimination from infected mice (Dornand et al., 2002; Dorneles et al., 2015).

The twin-arginine translocation (Tat) pathway exists in bacteria, archaea, and plants, and enables the transport of fully folded proteins across the cytoplasmic membrane. Tat substrates usually contain an N-terminal signal peptide with an S/TRRXFLK consensus motif (Palmer and Berks, 2012). Depending on the translocated substrates, the Tat pathway has been implicated in a number of cellular processes in bacteria, including cell motility (Ochsner et al., 2002), cell division (Stanley et al., 2001), biofilm formation (De Buck et al., 2005), iron and phosphate acquisition (Létoffé et al., 2009; Mickael et al., 2010), and resistance to heavy metals and antimicrobial peptides (Weatherspoon-Griffin et al., 2011). Our previous study showed that the Tat pathway is required for full *B*. *melitensis* M28 virulence in a macrophage cell model. Deletion of Tat leads to a higher sensitivity of bacteria to oxidative stress, and Tat mutants form defective cell envelopes, likely because cell wall/LPS biosynthesis-related substrates are not properly translocated (Yan et al., 2020). However, the effect of the Tat system on the macrophage immune response during *Brucella*-host interactions has not been extensively studied.

In this study, using label-free proteomics and parallel reaction monitoring, we sought to compare the dynamic proteomic responses of RAW264.7 macrophages to Tat mutants and the parental wild-type strain. We showed that compared to the response to wild-type infection, pattern recognition receptors and key inflammatory pathway-related proteins were significantly increased after Tat-mutant *Brucella* infection. Furthermore, Tat deficiency significantly induces proinflammatory cytokines and NO production. Finally, the contribution of NO production to Tat mutant *Brucella* survival in murine macrophages was explored.

## Materials and Methods

### Bacterial Strains and Culture Conditions

All strains and primers used in the study are listed in **Table S1**. *B. melitensis* M28 is a hypervirulent strain, and *B. melitensis* M28Δ*tatA* is an attenuated strain produced by homologous recombination. All studies involving live *B. melitensis* were performed under Harbin Veterinary Institute biosafety level 3 (BSL3) conditions. *B. melitensis* M28 and its derivatives were cultured on tryptic soy agar (TSA) or in tryptic soy broth (TSB) (Difco) at 37°C in a 5% CO_2_ atmosphere.

### Cell Culture and Infection

RAW264.7 (ATCC) murine macrophage cells were cultured in Dulbecco’s minimal essential medium (DMEM) (Gibco, USA) supplemented with 10% fetal bovine serum (FBS) without antibiotics and was incubated at 37°C in a 5% CO_2_ atmosphere. Measurement of the CFU counts within macrophages infected with *B. melitensis* M28 and its derivatives were described previously (Yan et al., 2020). For quantitative mass proteomics analysis, RAW264.7 cells (3×10^6^ cells per well) were seeded onto 6-well tissue culture plates and inoculated with bacterial culture diluted in DMEM (5% FBS) at an MOI (multiplicity of infection) of 100:1. After a 10-min centrifugation at 250×g, the plates were placed in a 5% CO_2_ atmosphere at 37°C for 3 h. Then, the cells were washed with DMEM three times and incubated in DMEM (5% FBS) containing 5 µg/mL gentamicin at 37°C and 5% CO_2_ for the indicated times. After 4 h, 24 h and 48 h, cells were collected, washed three times with ice-cold PBS and processed for label-free quantitative mass proteomic analysis.

### Protein Extraction and Trypsin Digestion

The M28-infected, M28Δ*tatA*-infected and mock-infected cells were sonicated three times on ice using a high-intensity ultrasonic processor (Scientz) in lysis buffer (8 M urea, 1% protease inhibitor cocktail). Debris was removed by centrifugation at 12,000 × g at 4°C for 10 min. Finally, the supernatant was collected, and the protein concentration was determined with a BCA kit according to the manufacturer’s instructions.

For digestion, the protein solution (300 μg for each sample) was reduced with 5 mM dithiothreitol for 30 min at 56°C and alkylated with 11 mM iodoacetamide for 15 min at room temperature in darkness. The protein sample was then diluted by adding 100 mM TEAB (Sigma-Aldrich, Saint Louis, USA) to urea (< 2 M). Finally, trypsin was added at a 1:50 trypsin: protein mass ratio for the first digestion overnight and a 1:100 trypsin: protein mass ratio for a second 4-h digestion.

### LC-MS/MS Analysis

The tryptic peptides were dissolved in solvent A (0.1% formic acid, 2% acetonitrile/in water) and directly loaded onto a homemade reversed-phase analytical column (25-cm length, 75-μm i.d.). Peptides were separated with a gradient from 6% to 24% solvent B (0.1% formic acid in acetonitrile) for 70 min, from 24% to 35% for 14 min and increasing to 80% in 3 min then holding at 80% for the final 3 min, all at a constant flow rate of 450 nL/min in a nanoElute UHPLC system (Bruker Daltonics).

The peptides were subjected to capillary electrophoresis followed by timsTOF Pro (Bruker Daltonics) mass spectrometry. The electrospray voltage applied was 1.60 kV. Precursors and fragments were analyzed with the TOF detector with an MS/MS scan range from 100 to 1700 m/z. The timsTOF Pro was operated in parallel accumulation serial fragmentation (PASEF) mode. Precursors with charge states from 0 to 5 were selected for fragmentation, and 10 PASEF-MS/MS scans were acquired per cycle. The dynamic exclusion was set to 30 s.

### Database Search and Bioinformatics Analysis

All MS raw files from the same batch were processed with MaxQuant (v1.6.6.0) and searched against the SwissProt *Mus musculus* protein database (version 2019.11, 17,032 sequences) concatenated with the reverse decoy database. Trypsin/P was specified as a cleavage enzyme and up to 2 missed cleavages and 5 modifications per peptide were allowed. The mass tolerance for precursor ions was set as 40 ppm for the first search and 40 ppm for the main search, and the mass tolerance for fragment ions was set as 0.04 Da. Carbamidomethylation on Cys was specified as a fixed modification and oxidation on Met and acetylation on the protein N-terminus were specified as variable modifications. The minimal peptide length was set to 7 residues. The false discovery rates (FDRs) of the peptides and proteins were set as 1%.

InterProScan software was used to annotate the function of the proteins by Gene Ontology (GO, http://www.ebi.ac.uk/interpro/) according to the protein sequence alignment method. Then, the proteins were classified based on the three GO annotation categories: biological process, cellular component and molecular function. Furthermore, functional enrichment analysis was performed on the basis of the GO and Kyoto Encyclopedia of Genes and Genomes (KEGG, http://www.genome.jp/kaas-bin/kaas_main) pathway analyses, and the corresponding functions and pathways with p < 0.05 (two-tailed Fisher’s exact test) were considered statistically significant.

### Quantification of Differentially Expressed Proteins (DEPs) by PRM

For the M28Δ*tatA*/M28-infected group, 15 upregulated DEPs were selected for validation by PRM. Protein extraction and tryptic digestion were performed following the same protocol used for the label-free quantitative proteomics experiment.

The tryptic peptides were dissolved in solvent A (0.1% formic acid in 2% acetonitrile) and directly loaded onto a homemade reversed-phase analytical column (15-cm length, 75-μm i.d.). The gradient increased from 6% to 22% with solvent B (0.1% formic acid in 90% acetonitrile) for 16 min, 22% to 32% for 6 min and increasing to 80% in 4 min then holding at 80% for the final 4 min, all at a constant flow rate of 450 nL/min in an EASY-nLC 1200 UPLC system.

PRM mass spectrometric analysis was performed on a HF-X mass spectrometer (Thermo, USA) coupled online to a UPLC. The electrospray voltage was applied at 2.1 kV. The m/z scan ranged from 378 to 1025 for a full scan, and intact peptides were detected in the Orbitrap at a resolution of 120,000. Peptides were then selected for MS/MS using an NCE setting of 28, and the fragments were detected in the Orbitrap at a resolution of 15,000. AGC was set at 3E6 for full MS and 1E5 for MS/MS. The maximum IT was set at 50 ms for full MS and 160 ms for MS/MS. The isolation window for MS/MS was set at 1.4 m/z. The resulting MS data were processed using Skyline (v.3.6) (Zhang et al., 2020). After normalizing the quantitative information, relative quantitative analysis was performed on the target peptides.

### RNA Extraction and Quantitative RT-PCR

Macrophage infection and RNA extraction were performed as previously described (Lei et al., 2015). Monolayers of RAW264.7 cells (~ 1.2×10^6^ cells per well) were infected with M28, M28Δ*tatA* and M28Δ*tatA* pBBR*tatA* at a multiplicity of infection (MOI) of 100. After a 10-min centrifugation at 250 × g, the plates were placed in a 5% CO_2_ atmosphere at 37°C for 3 h. Then, the cells were washed three times with DMEM and incubated in DMEM (5% FBS) containing 5 µg/mL gentamicin at 37°C and 5% CO_2_ for the indicated times. At 4 h, 24 h and 48 h post-infection, the supernatants were discarded, and macrophage total RNA was extracted by TRIzol reagent according to the instructions (Invitrogen). RNA was isolated from uninfected RAW264.7 cells and used as a negative control. RNA samples were treated to remove genomic DNA and subjected to reverse transcription using a PrimeScript RT reagent kit with gDNA Eraser (TaKaRa, Clontech). cDNA was used as the template for SYBR green-based qPCRs with TB Green Premix Ex Taq II reagent (TaKaRa, Clontech) and an ABI Quant 5 thermocycler. Fold changes in transcript levels were calculated using the threshold cycle (2^-ΔΔCt^) method (Pfaffl, 2001), and the levels were normalized according to β-actin expression.

### ELISA

The TNF-α and IL-6 secretion levels in each supernatant were determined by the ELISA kits (MultiSciences Biotech, China) according to the manufacturer’s instructions. These experiments were performed in triplicate with three biological repetitions.

### NO Measurement

To measure the amount of NO produced by macrophages, the macrophages were treated as indicated. Then, the culture supernatants were harvested by centrifugation and kept at -80 °C until analysis. The NO content of the culture supernatants was estimated by an analysis of nitrite accumulation with the Griess reaction as previously described (Bansal et al., 2009).

### Nitrosative Stress Susceptibility Assay

Resistance to nitrosative stress was assayed according to previously described protocols with some modifications (Kawaji et al., 2010; Dankai et al., 2016; Yan et al., 2020). A bacterial suspension from a single fresh colony was plated onto TSA plates and allowed to grow for 72 h. The cells were then harvested and resuspended in sterile phosphate-buffered saline (PBS) at an optical density at 600 nm (OD_600_) of ~ 0.02 (~ 1 × 10^8^ CFU/ml) before performing a 10-fold serial dilution. Five microliters of each dilution was spotted onto plain TSA plates and TSA plates supplemented with 5 mM NaNO_2_ (Sigma-Aldrich, St. Louis, USA). The plates were incubated at 37°C in a 5% CO_2_ atmosphere for 3 days to allow bacterial growth, which was followed by counting the CFU of each dilution. The ratio of CFU on the TSA plain plates (CFU_unstressed_) to CFU on the NaNO_2_-supplemented TSA plates (CFU_stressed_) was calculated for each dilution of the bacteria.

### Inhibition Assay

The NO synthases inhibitor L-NMMA (100 µM) was purchased from Selleck Chemicals (Houston, United States). Monolayers of RAW264.7 cells (~ 1.2×10^6^ cells per well) were pretreated with 100 µM L-NMMA for 1 h at 37°C. Then, the cells were inoculated with M28 or M28Δ*tatA* diluted in DMEM (5% FBS) at an MOI of 100:1. After a 10-min centrifugation at 250 × g, the plates were placed in a 5% CO_2_ atmosphere at 37°C for 3 h. Then, the cells were washed three times with DMEM and incubated in DMEM (5% FBS) containing 5 µg/mL gentamicin at 37°C and 5% CO_2_ for the indicated times. At the time of gentamicin addition, 100 µM L-NMMA was also added, and that point was defined as time 0. At 1, 24 and 48 hours postinfection, the infected cells in each well were washed three times with PBS and lysed with 1 ml of 0.1% Triton X-100 in PBS. The intracellular CFU counts were determined by plating serial dilutions on TSA with the appropriate antibiotics.

### Statistical Analysis

One-way ANOVA followed by Tukey’s test was used in cytokine production assays; for all other experiments, the Student’s *t* test was used to analyze differences between M28Δ*tatA* and the wild type (GraphPad, Prism). A *P* value of <0.05 was considered statistically significant.

## Results

### Differential Proteome Expression in RAW264.7 Cells After Wild-Type and Tat-Mutant *Brucella* Infection

**Figure 1:**
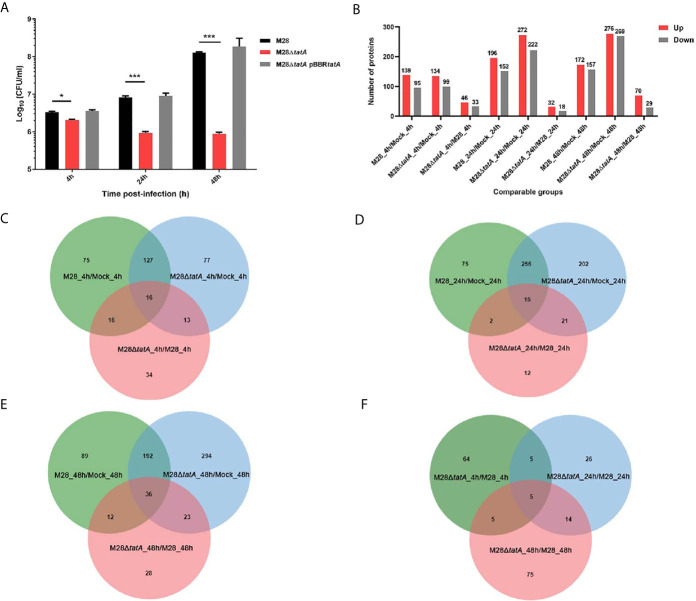
Label-free proteomics of RAW264.7 macrophages infected with M28 and M28Δ*tatA*. **(A)** Intracellular CFU of M28 and its derivatives in murine RAW264.7 cells. Asterisks denote statistically significant differences between the M28Δ*tatA* mutant group and the parental strain M28 group based on the Student’s *t* test (*p < 0.05; ***p < 0.001). All tests were done 3 times. **(B)** The number of DEPs in the different comparison groups. **(C)** Venn diagrams displaying the overlap of DEPs at 4 hpi. **(D)** Venn diagrams displaying the overlap of DEPs 24 hpi. **(E)** Venn diagrams displaying the overlap of DEPs 48 hpi. **(F)** For the M28Δ*tatA*/M28 group, Venn diagrams displaying the overlap of DEPs at 4, 24 and 48 hpi.

We began this study by comparing the intra-macrophage survival of the Tat mutant to that of the wild type (WT) at 4, 24, and 48 h postinfection (hpi). The results showed that at the early stage of infection (4 h) in murine RAW264.7 cells, deletion of *tatA* resulted in a 1.5-fold reduction in the number of intracellular CFUs, which became more severe at 24 h and 48 h postinfection (1-log and 2-log differences, respectively). Complementation of the M28Δ*tatA* strain with a plasmid-borne *tatA* gene restored the levels of bacterial survival to those of the wild type (**Figure 1A**). Then, we wanted to determine how host macrophages responded to Tat-mutant infection and compared it to the host macrophage response to wild-type *Brucella* infection. An LC-MS/MS-based label-free quantitative proteomics approach was used to assess the proteomic responses of RAW264.7 macrophages to wild-type M28 and M28Δ*tatA* mutant infections 4, 24 and 48 hpi. Uninfected RAW 264.7 macrophages were included as a control. In total, 6085 proteins were detected by proteomics analysis (A full list of identified proteins is available in **Supplementary Datasheet 2**). Significantly differentially produced proteins between the strain samples were identified using the criteria of a |fold change|>1.5 and a p value < 0.05 for peptide quantification. At all 3 time points, compared to the protein expression level after wild-type infection, dozens of proteins were differentially produced in the macrophages infected with the M28Δ*tatA* mutant (46 upregulated and 33 downregulated 4 hpi; 32 upregulated and 18 downregulated 24 hpi; 70 upregulated and 29 downregulated 48 hpi), with the number of upregulated proteins being higher than the number of downregulated proteins (**Figure 1B**), a full list of DEPs is available in **Supplementary Datasheet 3**.

A Venn diagram was created for each time point to show shared and unique differentially produced proteins among the 3 binary comparisons (**Figures 1C–E**). Additionally, shared and novel differentially produced proteins identified in the M28Δ*tatA*/M28 comparison at the 3 time points were analyzed with a Venn diagram, and the results demonstrated that five proteins, i.e., ISG15, SPP1, IFIT1, IFI202, and USP18, were uniformly upregulated in M28Δ*tatA*-infected macrophages at all 3 time points compared to those infected with the wild type (**Figure 1F**). Together, these results indicate that the proteomic responses of RAW264.7 macrophages to the M28Δ*tatA* mutant were different than their responses to wild-type strain infection throughout a 48-h course of infection.

### Functional Characterization of Differentially Expressed Proteins

To further understand the differential responses of macrophages to wild-type and Tat mutant *Brucella* infection, we performed GO classification and KEGG enrichment analysis on the DEPs as well as cluster analysis to identify the correlations of protein functions associated with the DEPs. These results are presented as heat maps (a full presentation of the results of GO classification and KEGG enrichment analysis is shown in **Figure S1**).

**Figure 2:**
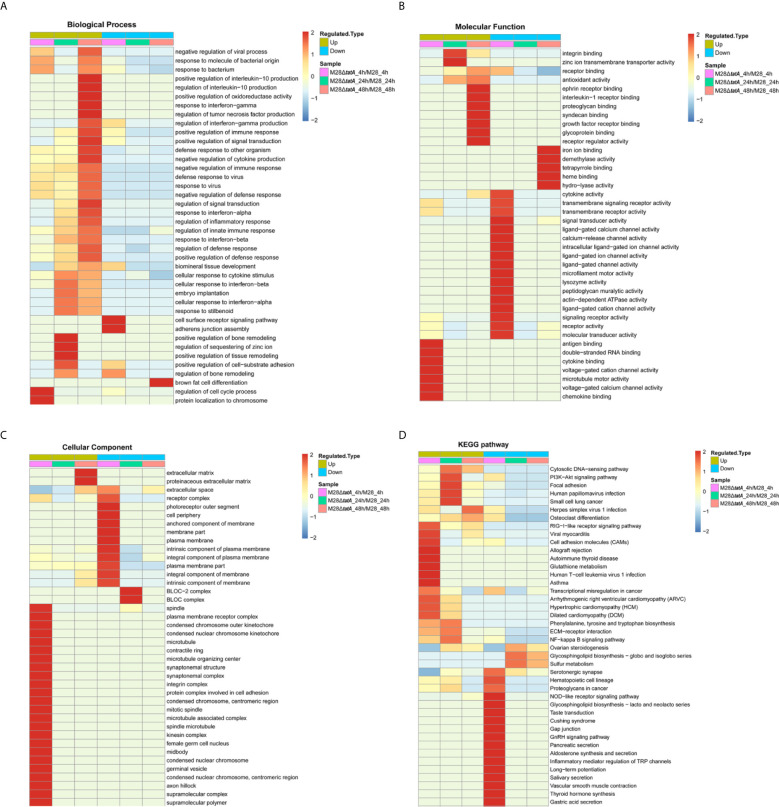
Functional enrichment analysis of DEPs in RAW264.7 cells after infection with M28 and M28Δ*tatA*. **(A–C)** Cluster analysis heat map based on GO enrichment. **(D)** Cluster analysis heat map based on KEGG enrichment. The horizontal direction represents the enrichment test results of the different parts, and the vertical direction is the description of the differential expression enrichment-related functions and KEGG pathways. Red indicates a strong degree of enrichment (the darker the red, the stronger the enrichment), and blue indicates a weaker enrichment (the lighter the blue, the weaker the enrichment).

GO annotation of the DEPs classified them into 3 categories: biological processes, molecular functions, and cellular components (Li et al., 2017). The host immune response represents one of the most important aspects during *Brucella*-macrophage interactions (He, 2012; Hop et al., 2018); thus, we focused on macrophage immune response-related proteins in this study. Remarkably, immune response-related proteins were mostly upregulated in M28Δ*tatA*-infected macrophages compared to those infected with the wild type. Specifically, these upregulated DEPs were mainly associated with the response to molecules of bacterial origin, the cellular response to cytokine stimulus and positive regulation of the immune response in the biological process category (**Figure 2A**); antigen binding, double-stranded RNA binding and cytokine binding in the molecular function category (**Figure 2B**); and plasma membrane receptor complex and condensed chromosome outer kinetochore in the cellular component category (**Figure 2C**). These results suggest that the Tat mutant differs from the wild-type strain in eliciting a host immune response.

Then, KEGG pathway annotation and enrichment analysis were performed to determine the signaling pathways associated with upregulated DEPs. In the M28Δ*tatA*/M28 group, the DEPs were mainly enriched in the cytosolic DNA-sensing pathway, P13K-Akt signaling pathway, RIG-I-like receptor signaling pathway and NF-*κ*B signaling pathway (**Figure 2D**). Therefore, these results provide additional evidence supporting the hypothesis that macrophages infected with the Tat-mutant strain exhibit different immune responses than those infected with wild-type *Brucella*.

### Pattern Recognition Receptor-Related Proteins Were Upregulated in the Tat Mutant/WT Group

GO and KEGG enrichment analysis indicated that the Tat mutant differs from the wild-type strain in eliciting host immune responses. Pattern recognition receptors (PRRs), including Toll-like receptors (TLRs), NOD-like receptors (NLRs), AIM2-like receptors (ALRs), RIG-I-like receptors (RLRs), C-type lectin receptors (CLRs) and Sequestosome 1/p62-like receptors (SLRs), provide the first line of defense against both extracellular and intracellular pathogens (Kumar et al., 2013; Kaakoush et al., 2015). Thus, we hypothesized that the Tat mutant could be recognized by macrophages in a different way than the wild type, leading to different macrophage immune responses.

**Figure 3:**
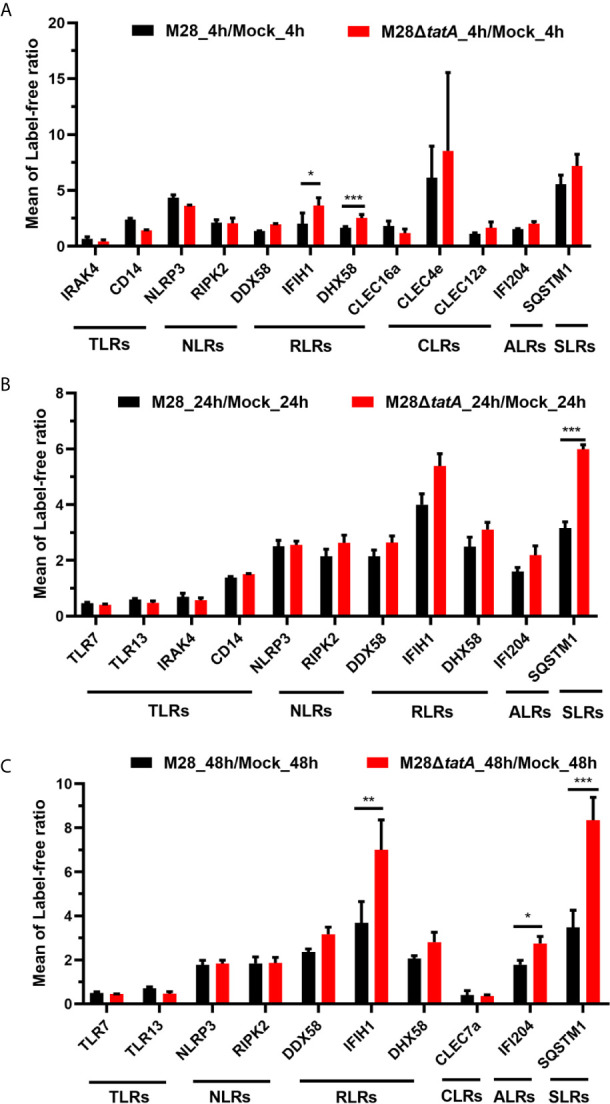
Regulation of proteins associated with pattern recognition receptors in RAW264.7 cells after infection with M28 and M28Δ*tatA* at 4 hpi **(A)**, 24 hpi **(B)** and 48 hpi **(C)**. Student’s *t* test was used to evaluate significant differences between the M28Δ*tatA* mutant and the wild type. *p < 0.05; **p < 0.01; ***p < 0.001.

We identified several RLRs, ALRs and SLRs that were altered upon Tat mutant *Brucella* infection, and four of them were significantly upregulated (**Figure 3**). Most of the upregulated proteins, including DHX58, IFIH1, and IFI204, belong to the functional category of cytosolic nucleic acid sensing receptors, suggesting the importance of this class in recognizing Tat mutant infection (**Figures 3A, C**). In addition, the SLR family protein SQSTM1 was also significantly upregulated upon Tat mutant *Brucella* infection at 24 and 48 hpi (**Figures 3B, C**), suggesting that autophagy may play a role in mediating host recognition of the Tat mutant. In summary, these data suggest that PRRs, especially RLR-related proteins, may play a more important role in the recognition of Tat mutant infection by macrophages than in recognition of the wild type.

### Key Inflammatory Pathway-Related Proteins Were Upregulated in the Tat Mutant/WT Group

**Figure 4:**
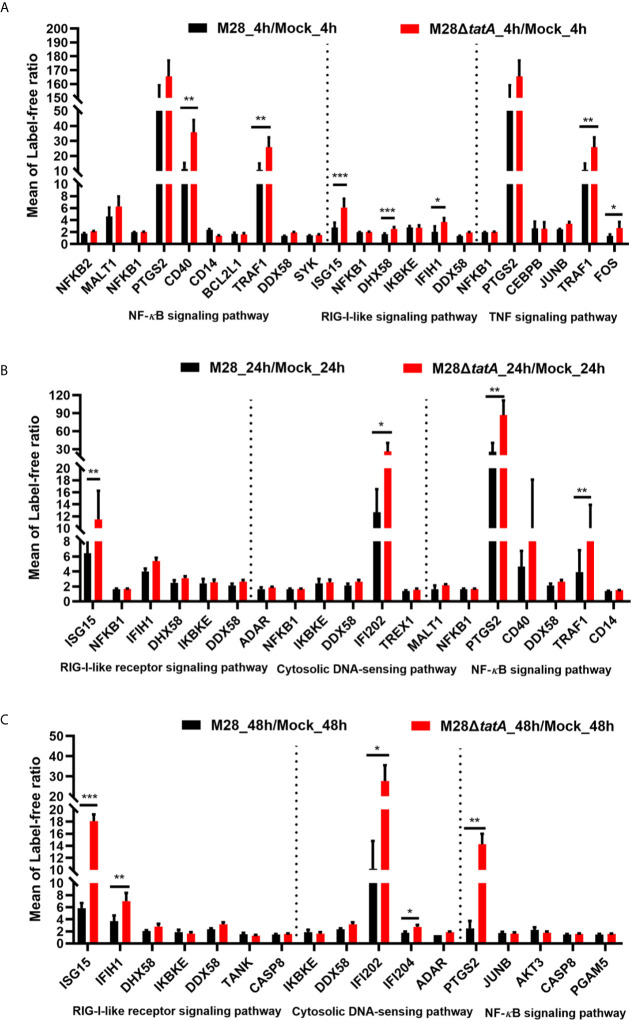
Regulation of proteins involved in key inflammatory pathways in RAW264.7 cells after infection with M28 and M28Δ*tatA* at 4 hpi **(A)**, 24 hpi **(B)** and 48 hpi **(C)**. Student’s *t* test was used to evaluate significant differences between the M28Δ*tatA* mutant and the wild type. *p < 0.05; **p < 0.01; ***p < 0.001.

After PRRs bind to distinct ligands, specific signal transduction pathways are activated, resulting in the induction of numerous cytokine and chemokine genes (Gomes et al., 2012; Kipanyula et al., 2013). Based on the results of the KEGG pathway enrichment, we analyzed proteins within key inflammatory pathways that were significantly upregulated following Tat mutant *Brucella* infection of murine RAW264.7 macrophages. We identified 10 differentially expressed proteins associated with the RIG-I-like signaling pathway, cytosolic DNA-sensing signaling pathway, NF-*κ*B signaling pathway and TNF signaling pathway (**Figure 4**). Half of these 10 proteins are involved in the cytosolic nucleic acid signaling pathways, such as IFIH1, DHX58 and ISG15 in the RIG-I-like signaling pathway and IFI204 and IFI202 in the cytosolic DNA-sensing signaling pathway, suggesting the importance of this pathway in the response to Tat mutant infection.

The NF-*κ*B signaling pathway is strongly induced downstream of most PRRs, including TLRs, NLRs and RLRs (Kipanyula et al., 2013; Kumar et al., 2013). As shown in **Figure 4**, NF-*κ*B signaling pathway-related proteins such as PTGS2, CD40 and TRAF1 were significantly upregulated after Tat mutant *Brucella* infection, especially PTGS2, suggesting that PTGS2, which is a key mediator of inflammation (Kipanyula et al., 2013; Gagnaire et al., 2016), may play more important roles in the Tat mutant-induced immune response of macrophages than in the M28-induced response. We also identified TNF signaling pathway-related proteins, such as TRAF1, FOS and PTGS2, that were significantly upregulated, and these results are consistent with previous reports that the TNF signaling pathway in macrophages is involved in *Brucella* infection (Caron et al., 1996; Hop et al., 2017).

Taken together, these data suggest that key inflammatory pathways, especially the cytosolic nucleic acid signaling pathway and NF-*κ*B signaling pathway-related proteins, may play a more important role in the immune response of Tat-mutant infection by macrophages than in the immune response of the wild type.

### PRM Validation of the DEPs Identified in the Proteomic Analysis

To verify the differentially expressed proteins in the label-free quantitative proteomics analysis, LC-PRM was applied to analyze the candidate peptides of the 15 target proteins in the M28Δ*tatA*/M28 groups identified on the basis of the upregulated DEPs in the GO and KEGG databases combined with the functional annotation information. The relative abundance of the peptides from the individual proteins was determined and normalized according to the corresponding total peak area (Gao et al., 2019; Zhang et al., 2020). As shown in **Table 1**, cytosolic nucleic acid signaling pathway-related proteins such as IFIH1, ISG15 and IFI202, and NF-*κ*B signaling pathway-related proteins such as PTGS2, CD40 and TRAF1, exhibited fold-changes in expression similar to those shown in the label-free proteomics. The results obtained by proteomic analysis and PRM were consistent with each other and confirmed that the expression of macrophage immune-related proteins was significantly increased after Tat-mutant *Brucella* infection compared to that induced by wild-type strain infection.

**Table 1:** PRM validation of the proteomics.

Protein accession	Coding genes	Mean of PRM/Label-free ratio		
		M28Δ*tatA*_4h/M28_4h	M28Δ*tatA*_24h/M28_24h	M28Δ*tatA*_48h/M28_48h
P27512	CD40	2.61**/3.16**	–	–
Q64339	ISG15	2.29**/2.21**	2.06*/1.71**	3.06*/3.09**
Q8R5F7	IFIH1	1.59*/1.93*	–	1.70*/1.81**
Q9WTV6	USP18	1.30/1.70*	–	2.15**/2.75**
P39428	TRAF1	1.46*/2.47**	–	–
Q9R002	IFI202	1.53**/1.75**	1.46/2.09*	4.08*/3.23*
P54987	ACOD1	–	1.78**/2.00**	2.53*/2.33**
Q05769	PTGS2	–	3.88**/3.47**	5.65**/6.79**
Q64337	SQSTM1	–	2.32**/1.90**	3.05**/2.42**
Q64345	IFIT3	–	2.12**/3.29*	3.74**/11.09*
Q8BPX9	SLC15A3	1.59/1.25	2.11**/2.28*	3.11**/4.41**
Q60766	IRGM1	–	1.62**/1.54**	–
P10923	SPP1	–	1.91*/1.66**	3.31**/3.96**
Q9QY24	ZBP1	–	–	2.85/6.08**
O08573	LGALS9	–	–	2.10**/2.97**

-, not significantly regulated, *p ＜ 0.05, **p ＜ 0.01.

### Tat-Mutant Infection Induces Higher Proinflammatory Cytokine and NO Production in RAW264.7 Macrophages Than WT Infection

**Figure 5:**
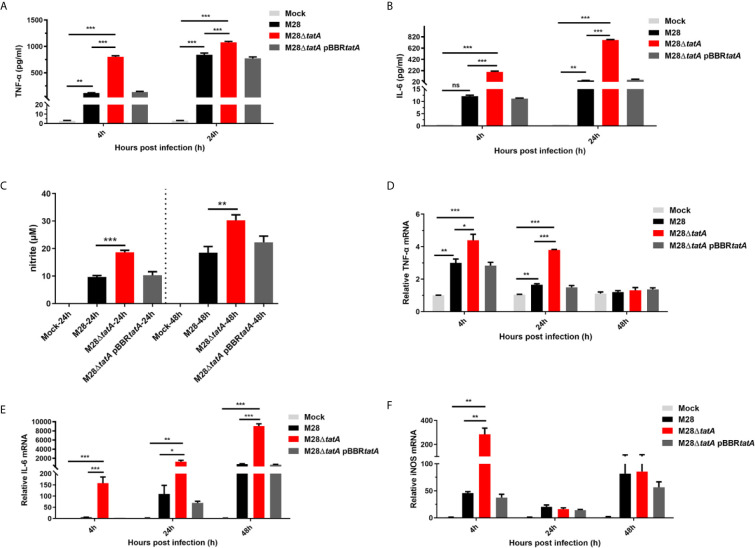
Expression and production of nitric oxide (NO) and inflammatory cytokines in RAW264.7 cells after infection with M28, M28Δ*tatA*, and M28Δ*tatA* pBBR*tatA*. The production of TNF-α **(A)** and IL-6 **(B)** in RAW264.7 cells after infection with the indicated strain was determined by ELISA. The production of NO **(C)** in RAW264.7 cells after infection with the indicated strains was determined by Griess assays. The relative transcription levels of TNF-α **(D)**, IL-6 **(E)** and iNOS **(F)** in RAW264.7 cells after infection with the indicated strain were determined by qRT-PCR. The SD is indicated by the error bar. *p < 0.05; **p < 0.01; ***p < 0.001; ns, not significant.

To gain more insights into the altered immune response in M28Δ*tatA*-infected macrophages, we examined the production of proinflammatory cytokines (TNF-α and IL-6) and NO relevant to host immune responses to *Brucella* infection. At 4 and 24 hpi, the production of the proinflammatory cytokines TNF-α and IL-6 in macrophages infected by *Brucella* was greatly increased compared to that produced in uninfected cells; however, the M28Δ*tatA-*infected macrophages produced significantly higher levels of these cytokines than the wild-type-infected cells (p<0.001) (**Figures 5A, B**).

NO is an important immune mediator by which macrophages control brucellosis (López-Urrutia et al., 2000). Culture supernatants of RAW264.7 cells were assayed for NO production by the Griess assay 24 h and 48 h after infection with the wild-type or Tat-mutant strains. We found that the production of NO in the M28Δ*tatA-*infected group was significantly higher than that in the M28-infected group (p<0.01) (**Figure 5C**). Notably, throughout the 24-h course of M28Δ*tatA* infection, the expression trends of the TNF-α, IL-6 and inducible nitric oxide synthase (iNOS) genes were in line with the ELISA results (**Figures 5D–F**). These findings suggest that, compared to the response to infection with wild-type M28, infection with the Tat mutant elicited stronger immune-stimulating activity in RAW264.7 cells, as evidenced by a higher production of TNF-α, IL-6, and NO.

### NO Production in Macrophages Contributes to Restricting Intracellular Survival of Tat Mutants

Evidence of NO-dependent antimicrobial activity by murine macrophages against *Brucella* is available (Gross et al., 1998; López-Urrutia et al., 2000). Compared to wild-type infection, Tat mutant infection significantly induced iNOS transcription and NO production in RAW264.7 macrophages. We thus hypothesized that elevated NO production contributes to reducing the Tat mutant load within macrophages.

**Figure 6:**
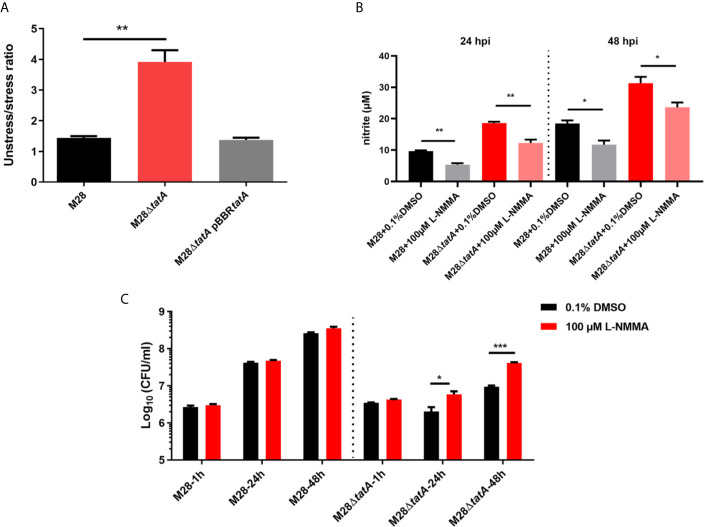
Sensitivity of various *B. melitensis* M28 strains to nitrosative stress and inhibition of NO production increases the intracellular survival of Tat mutant *Brucella*. **(A)** Nitrosative stress survival assay. Serially diluted cultures of the wild type, M28Δ*tatA* mutant, and complemented strain were spotted on plain TSA plates or TSA plates containing NaNO_2_ (5 mM). After 72 h of growth at 37°C under 5% CO_2_, CFUs were counted, and the ratio of CFUs on the TSA plain plates (CFU_unstressed_) to CFUs on the NaNO_2_-supplemented TSA plates (CFU_stressed_) was calculated for each strain. **(B)** L-NMMA treatment reduced NO production in M28- and M28Δ*tatA*-infected RAW264.7 cells. **(C)** L-NMMA treatment increased Tat mutant *Brucella* intracellular survival in RAW264.7 cells at 24 h and 48 h post infection; the data are shown as the mean ± standard error of the mean. Student’s *t* test was used to evaluate significant differences between the M28Δ*tatA* mutant and the wild type. *p < 0.05; **p < 0.01; ***p < 0.001.

First, we determined whether the M28Δ*tatA* mutant is more sensitive to nitrosative stress. Upon treatment with 5 mM NaNO_2_ on tryptic soy agar (TSA) plates, the bacterial CFU of M28Δ*tatA* was decreased by ~3.6-fold, whereas that of the wild type was decreased by only ~1.4-fold (p<0.01; **Figure 6A**), indicating that the M28Δ*tatA* mutant is more sensitive to NaNO_2_ than the wild type. L-NMMA is a selective inhibitor of NO synthases, and it can reduce the production of NO during M28 and M28Δ*tatA* infection of macrophages (**Figure 6B**). **Figure 6C** shows that at 24 and 48 hpi, the intracellular M28Δ*tatA* load was higher in the presence of L-NMMA than in the absence of L-NMMA (p < 0.05), whereas the addition of L-NMMA did not affect the intracellular M28 load at any of the 3 time points. Collectively, these results suggest that NO production in macrophages contributes to restricting the intracellular survival of Tat mutants.

## Discussion

The Tat protein export system is located in the cytoplasmic membranes of many bacteria and has the highly unusual ability to transport fully folded proteins. We have previously shown that a Tat mutant of *Brucella* had significantly reduced virulence in macrophages. This decrease in virulence can be at least partially explained by the impaired antistress ability of the Tat mutants. However, inactivation of Tat led to envelope defects and consequently could expose certain cellular components to macrophages during infection, which may elicit differential host immune responses. In this study, using label-free quantitative proteomics, we present the global proteomic analysis of RAW264.7 macrophage responses to wild-type and Tat-mutant *Brucella* throughout the course of infection. Indeed, with a combination of quantitative proteomics, ELISA and Griess reaction assays, we revealed that Tat system deficiency significantly upregulated PRRs and key inflammatory pathway-related proteins compared to the wild type. To our knowledge, this is the first analysis of host responses to Tat mutant infection on a global scale, and this work greatly improves our understanding of the role of Tat in *Brucella* virulence.

Comparing the M28 24h group and M28 4h group, where bacterial load was higher at 24 h, we found that proteins including IFIH1, DDX58, DHX58 and ISG15 were significantly induced at 24 h, suggesting that expression levels of these proteins positively correlated with *Brucella* bacterial load. However, comparing M28Δ*tatA* with WT, where M28Δ*tatA* was present in lower numbers than the WT, these proteins were still induced, suggesting that changes in protein expression was largely due to the mutation of Tat, which likely overrode the bacterial load difference between M28Δ*tatA* and WT. As another support of this notion, similar trends were observed for cytokine production. We showed that cytosolic nucleic acid signaling pathway-related proteins such as IFIH1 (MDA5), ISG15, IFI202 and IFI204 were significantly upregulated in the M28Δ*tatA*/M28 infection group. The entry of bacterial nucleic acids into the cytosol of infected cells is essential for the generation of antimicrobial immunity (Abdullah et al., 2012). As a member of the RIG-I-like receptor (RLR) family, MDA5 is an important receptor of cytosolic RNA (Yoneyama et al., 2005). Upon engagement with nonself nucleic acids, RLRs activate serine kinase signaling cascades that converge on interferon regulatory factor (IRF) and NF-*κ*B transcription factors, resulting in the expression of type I IFN, chemokines (CCL2/3/5) and inflammatory cytokines (TNF-α and IL-6) (Pollpeter et al., 2011; Kipanyula et al., 2013). *Brucella* infection markedly induces the expression and secretion of TNF-α, which, in turn, binds to TNFR-1, further activating the NF-*κ*B signaling pathway, stimulating macrophages to produce NO and ROS, and eliminating intracellular bacteria (Hop et al., 2017). Additionally, a recent study showed that endogenous IL-6 can bind to its receptor complex IL-6R/Gp130 and thus elicit activation of the JAK2/STAT pathway, promoting the production of effectors of phagolysosome maturation-trafficking regulators and lysosomal enzymes, as well as proinflammatory and anti-inflammatory cytokines that facilitate the clearance of *Brucella* infection in macrophages (Hop et al., 2019). Our results showed that Tat-mutant infection can significantly induce the expression of TNF-α and IL-6 (**Figure 5**). Therefore, upregulation of the immunosurveillance pathway (RIG-I/MDA5 pathway) and increased production of TNF-α and IL-6 may constitute a mechanism underlying the restriction of Tat mutant survival by macrophages.

Regulation of the nitric oxide synthase biosynthetic process was significantly enriched in the M28Δ*tatA*/M28 group (**Figure S2**). iNOS is an enzyme catalyzing NO production, which is induced by TNF-α and IL-1β, among other inflammatory factors, and NO is an essential mediator of macrophage cytotoxicity against a variety of microorganisms (Bansal et al., 2009; Kipanyula et al., 2013). Additionally, NO can increase the expression of some inflammatory response proteins, such as PTGS2 (COX-2) and TNF-α, through NF-*κ*B activation (Dey and Bishayi, 2017). While iNOS produces antibacterial NO, COX-2 converts arachidonic acid to prostaglandins (PGs). After synthesis, PGs are secreted from macrophage cells and function as autocrine agents to regulate macrophage functions, including the production of proinflammatory cytokines and NO (Shi et al., 2009). The induction of NO and COX-2 in macrophages by pathogenic microbes and the roles of these regulatory molecules in controlling microbial infection are well documented (López-Urrutia et al., 2000; Bowman and Bost, 2004; Bernard and Gallo, 2010; Ren et al., 2020). Notably, the ability of Tat-mutant *Brucella* to resist nitrite *in vitro* was significantly lower than that of the wild-type strain (**Figure 6A**). Indeed, COX-2 and NO were significantly induced, and inhibition of NO and COX-2 significantly increased the intracellular survival of Tat mutant *Brucella* (**Figures S3** and **6C**). Therefore, elevated production of NO may serve as another mechanism used by macrophages to restrict intracellular growth of the Tat mutant.

Although we showed that the Tat mutant markedly alters immunity-related pathways and that increased production of NO could contribute to restricted survival of the Tat mutant in macrophages, it remains unknown which Tat substrate(s) is responsible for the altered immune responses in macrophages. It is tempting to assume that several substrates involved in cell envelope biosynthesis, including two murein L,D-transpeptidases and an O-antigen/exopolysaccharide biosynthesis protein (Yan et al., 2020), could be contributing factors during *Brucella*-host cell interactions. In addition, defects in LPS or other surface structures may allow detection by macrophages and subsequently lead to a stronger host immune response, as structures such as the core of LPS serve as a shield to evade innate immunity recognition (Conde-Alvarez et al., 2012). More experiments are needed to address these issues.

Overall, our study presents the first application of quantitative proteomics to identify RAW264.7 macrophage proteins differentially expressed between infections with a *Brucella* Tat mutant and the wild-type strain. Infection with Tat-mutant *Brucella* significantly upregulated PRRs and key inflammatory pathway-related proteins compared with the wild type. Our work has provided insights into *Brucella*-host cell interactions and may shed light on approaches to designing live attenuated vaccines.

## Data Availability Statement

The datasets presented in this study can be found in online repositories. The names of the repository/repositories and accession number(s) can be found below: https://www.ebi.ac.uk/pride/archive/, PXD024658.

## Author Contributions

XY, WC, GL and ZB conceived and designed the experiments. XY, SH and YY performed the experiments. XY, WL and DX analyzed the data. XY, GL, WC and ZB wrote and reviewed the paper. All authors participated in discussions of the results. ZB, WC, and GL provided the resources and the funding. All authors contributed to the article and approved the submitted version.

## Funding

This work was supported by The National Key Research and Development Program of China (2018YFD0500501), Sci-tech innovation project of Chinese Academy of Agricultural Sciences (2017-2020) and PI Startup Funding of Harbin Veterinary Research Institute (2017-2020).

## Conflict of Interest

The authors declare that the research was conducted in the absence of any commercial or financial relationships that could be construed as a potential conflict of interest.

## References

[B1] Abdullah Z. Schlee M. Roth S. Mraheil M. A. Barchet W. Böttcher J.. (2012). RIG-I Detects Infection With Live *Listeria* by Sensing Secreted Bacterial Nucleic Acids. EMBO J. 31 (21), 4153–4164. 10.1038/emboj.2012.274 23064150 PMC3492734

[B2] Arenas G. N. Staskevich A. S. Aballay A. Mayorga L. S. (2000). Intracellular Trafficking of *Brucella Abortus* in J774 Macrophages. Infect. Immun. 68 (7), 4255–4263. 10.1128/iai.68.7.4255-4263.2000 10858243 PMC101738

[B3] Bansal K. Narayana Y. Patil S. A. Balaji K. N. (2009). *M. Bovis* Bcg Induced Expression of Cox-2 Involves Nitric Oxide-Dependent and -Independent Signaling Pathways. J. Leukoc. Biol. 85 (5), 804–816. 10.1189/jlb.0908561 19228814

[B4] Bernard J. J. Gallo R. L. (2010). Cyclooxygenase-2 Enhances Antimicrobial Peptide Expression and Killing of *Staphylococcus Aureus* . J. Immunol. 185 (11), 6535–6544. 10.4049/jimmunol.1002009 20971925 PMC3025174

[B5] Bowman C. C. Bost K. L. (2004). Cyclooxygenase-2-Mediated Prostaglandin E2 Production in Mesenteric Lymph Nodes and in Cultured Macrophages and Dendritic Cells After Infection With *Salmonella* . J. Immunol. 172 (4), 2469–2475. 10.4049/jimmunol.172.4.2469 14764719

[B6] Byndloss M. X. Tsolis R. M. (2016). *Brucella* Spp. Virulence Factors and Immunity. Annu. Rev. Anim. Biosci. 4, 111–127. 10.1146/annurev-animal-021815-111326 26734887

[B7] Caron E. Gross A. Liautard J. P. Dornand J. (1996). *Brucella* Species Release a Specific, Protease-Sensitive, Inhibitor of TNF-Alpha Expression, Active on Human Macrophage-Like Cells. J. Immunol. 156 (8), 2885–2893. 8609408

[B8] Celli J. (2006). Surviving Inside a Macrophage: The Many Ways of *Brucella* . Res. Microbiol. 157 (2), 93–98. 10.1016/j.resmic.2005.10.002 16364608

[B9] Conde-Alvarez R. Arce-Gorvel V. Iriarte M. Mancek-Keber M. Barquero-Calvo E. Palacios-Chaves L.. (2012). The Lipopolysaccharide Core of *Brucella Abortus* Acts as a Shield Against Innate Immunity Recognition. PloS Pathog. 8 (5). 10.1371/journal.ppat.1002675 PMC3349745 22589715

[B10] Dankai W. Pongpom M. Youngchim S. Cooper C. R. Jr. Vanittanakom N. (2016). The Yapa Encodes Bzip Transcription Factor Involved in Stress Tolerance in Pathogenic Fungus *Talaromyces Marneffei* . PloS One 11 (10), e0163778. 10.1371/journal.pone.0163778 27706212 PMC5051730

[B11] De Buck E. Maes L. Meyen E. Van Mellaert L. Geukens N. Anne J.. (2005). *Legionella Pneumophila* Philadelphia-1 Tatb and Tatc Affect Intracellular Replication and Biofilm Formation. Biochem. Biophys. Res. Commun. 331 (4), 1413–1420. 10.1016/j.bbrc.2005.04.060 15883032

[B12] Dey S. Bishayi B. (2017). Effect of Inos Inhibitor LNMMA Along With Antibiotics Chloramphenicol or Ofloxacin in Murine Peritoneal Macrophages Regulates *S. Aureus* Infection as Well as Inflammation: An In Vitro Study. Microb. Pathog. 105, 307–320. 10.1016/j.micpath.2017.02.031 28242423

[B13] Dornand J. Gross A. Lafont V. Liautard J. Oliaro J. Liautard J. P. (2002). The Innate Immune Response Against *Brucella* in Humans. Vet. Microbiol. 90 (1-4), 383–394. 10.1016/s0378-1135(02)00223-7 12414158

[B14] Dorneles E. M. Teixeira-Carvalho A. Araújo M. S. Sriranganathan N. Lage A. P. (2015). Immune Response Triggered by *Brucella Abortus* Following Infection or Vaccination. Vaccine 33 (31), 3659–3666. 10.1016/j.vaccine.2015.05.057 26048781

[B15] Gagnaire A. Gorvel L. Papadopoulos A. Von Bargen K. Mège J. L. Gorvel J. P. (2016). Cox-2 Inhibition Reduces *Brucella* Bacterial Burden in Draining Lymph Nodes. Front. Microbiol. 7:1987. 10.3389/fmicb.2016.01987 28018318 PMC5149544

[B16] Gao X. Guo X. Li M. Jia H. Lin W. Fang L.. (2019). Interleukin 8 and Pentaxin (C-Reactive Protein) as Potential New Biomarkers of Bovine Tuberculosis. J. Clin. Microbiol. 57 (10). 10.1128/jcm.00274-19 PMC6760949 31340991

[B17] Gomes M. T. Campos P. C. de Almeida L. A. Oliveira F. S. Costa M. M. Marim F. M.. (2012). The Role of Innate Immune Signals in Immunity to *Brucella Abortus* . Front. Cell Infect. Microbiol. 2, 130. 10.3389/fcimb.2012.00130 23112959 PMC3480720

[B18] Gross A. Spiesser S. Terraza A. Rouot B. Caron E. Dornand J. (1998). Expression and Bactericidal Activity of Nitric Oxide Synthase in *Brucella Suis*-Infected Murine Macrophages. Infect. Immun. 66 (4), 1309–1316. 10.1128/iai.66.4.1309-1316.1998 9529047 PMC108054

[B19] He Y. (2012). Analyses of *Brucella* Pathogenesis, Host Immunity, and Vaccine Targets Using Systems Biology and Bioinformatics. Front. Cell Infect. Microbiol. 2, 2. 10.3389/fcimb.2012.00002 22919594 PMC3417401

[B20] He Y. Reichow S. Ramamoorthy S. Ding X. Lathigra R. Craig J. C.. (2006). *Brucella Melitensis* Triggers Time-Dependent Modulation of Apoptosis and Down-Regulation of Mitochondrion-Associated Gene Expression in Mouse Macrophages. Infect. Immun. 74 (9), 5035–5046. 10.1128/iai.01998-05 16926395 PMC1594834

[B21] Hop H. T. Arayan L. T. Huy T. X. N. Reyes A. W. B. Vu S. H. Min W.. (2018). The Key Role of C-Fos for Immune Regulation and Bacterial Dissemination in *Brucella* Infected Macrophage. Front. Cell Infect. Microbiol. 8, 287. 10.3389/fcimb.2018.00287 30186773 PMC6110913

[B22] Hop H. T. Huy T. X. N. Reyes A. W. B. Arayan L. T. Vu S. H. Min W.. (2019). Interleukin 6 Promotes *Brucella Abortus* Clearance by Controlling Bactericidal Activity of Macrophages and CD8(+) T Cell Differentiation. Infect. Immun. 87 (11). 10.1128/iai.00431-19 PMC6803322 31451617

[B23] Hop H. T. Reyes A. W. B. Huy T. X. N. Arayan L. T. Min W. Lee H. J.. (2017). Activation of NF-Kb-Mediated TNF-Induced Antimicrobial Immunity is Required for the Efficient *Brucella Abortus* Clearance in RAW 264.7 Cells. Front. Cell Infect. Microbiol. 7, 437. 10.3389/fcimb.2017.00437 29062811 PMC5640714

[B24] Jiang X. Baldwin C. L. (1993). Effects of Cytokines on Intracellular Growth of *Brucella Abortus* . Infect. Immun. 61 (1), 124–134. 10.1128/iai.61.1.124-134.1993 8418034 PMC302696

[B25] Jiang X. Leonard B. Benson R. Baldwin C. L. (1993). Macrophage Control of *Brucella Abortus*: Role of Reactive Oxygen Intermediates and Nitric Oxide. Cell Immunol. 151 (2), 309–319. 10.1006/cimm.1993.1241 8402938

[B26] Kaakoush N. O. Deshpande N. P. Man S. M. Burgos-Portugal J. A. Khattak F. A. Raftery M. J.. (2015). Transcriptomic and Proteomic Analyses Reveal Key Innate Immune Signatures in the Host Response to the Gastrointestinal Pathogen *Campylobacter Concisus* . Infect. Immun. 83 (2), 832–845. 10.1128/iai.03012-14 25486993 PMC4294260

[B27] Kawaji S. Zhong L. Whittington R. J. (2010). Partial Proteome of *Mycobacterium Avium* Subsp. Paratuberculosis Under Oxidative and Nitrosative Stress. Vet. Microbiol. 145 (3-4), 252–264. 10.1016/j.vetmic.2010.03.025 20413229

[B28] Kipanyula M. J. Seke Etet P. F. Vecchio L. Farahna M. Nukenine E. N. Nwabo Kamdje A. H. (2013). Signaling Pathways Bridging Microbial-Triggered Inflammation and Cancer. Cell Signal 25 (2), 403–416. 10.1016/j.cellsig.2012.10.014 23123499

[B29] Kumar S. Ingle H. Prasad D. V. Kumar H. (2013). Recognition of Bacterial Infection by Innate Immune Sensors. Crit. Rev. Microbiol. 39 (3), 229–246. 10.3109/1040841x.2012.706249 22866947

[B30] Lei S. Zhong Z. Ke Y. Yang M. Xu X. Ren H.. (2015). Deletion of the Small Rna Chaperone Protein Hfq Down Regulates Genes Related to Virulence and Confers Protection Against Wild-Type *Brucella* Challenge in Mice. Front. Microbiol. 6, 1570. 10.3389/fmicb.2015.01570 26834720 PMC4718986

[B31] Létoffé S. Heuck G. Delepelaire P. Lange N. Wandersman C. (2009). Bacteria Capture Iron From Heme by Keeping Tetrapyrrol Skeleton Intact. Proc. Natl. Acad. Sci. U. S. A. 106 (28), 11719–11724. 10.1073/pnas.0903842106 19564607 PMC2710666

[B32] Li P. Wang R. Dong W. Hu L. Zong B. Zhang Y.. (2017). Comparative Proteomics Analysis of Human Macrophages Infected With Virulent *Mycobacterium Bovis* . Front. Cell Infect. Microbiol. 7, 65. 10.3389/fcimb.2017.00065 28337427 PMC5343028

[B33] López-Urrutia L. Alonso A. Nieto M. L. Bayón Y. Orduña A. Sánchez Crespo M. (2000). Lipopolysaccharides of *Brucella Abortus* and *Brucella Melitensis* Induce Nitric Oxide Synthesis in Rat Peritoneal Macrophages. Infect. Immun. 68 (3), 1740–1745. 10.1128/iai.68.3.1740-1745.2000 10679001 PMC97342

[B34] Mickael C. S. Lam P. K. Berberov E. M. Allan B. Potter A. A. Koster W. (2010). *Salmonella Enterica* Serovar Enteritidis Tatb and Tatc Mutants are Impaired in Caco-2 Cell Invasion In Vitro and Show Reduced Systemic Spread in Chickens. Infect. Immun. 78 (8), 3493–3505. 10.1128/IAI.00090-10 20498258 PMC2916284

[B35] Ochsner U. A. Snyder A. Vasil A. I. Vasil M. L. (2002). Effects of the Twin-Arginine Translocase on Secretion of Virulence Factors, Stress Response, and Pathogenesis. Proc. Natl. Acad. Sci. U. S. A. 99 (12), 8312–8317. 10.1073/pnas.082238299 12034867 PMC123064

[B36] Palmer T. Berks B. C. (2012). The Twin-Arginine Translocation (TAT) Protein Export Pathway. Nat. Rev. Microbiol. 10 (7), 483–496. 10.1038/nrmicro2814 22683878

[B37] Pfaffl M. W. (2001). A New Mathematical Model for Relative Quantification in Real-Time RT-PCR. Nucleic Acids Res. 29 (9), e45. 10.1093/nar/29.9.e45 11328886 PMC55695

[B38] Pollpeter D. Komuro A. Barber G. N. Horvath C. M. (2011). Impaired Cellular Responses to Cytosolic DNA or Infection With *Listeria Monocytogenes* and Vaccinia Virus in the Absence of the Murine LGP2 Protein. PloS One 6 (4), e18842. 10.1371/journal.pone.0018842 21533147 PMC3077416

[B39] Ren H. Chen X. Jiang F. Li G. (2020). Cyclooxygenase-2 Inhibition Reduces Autophagy of Macrophages Enhancing Extraintestinal Pathogenic *Escherichia Coli* Infection. Front. Microbiol. 11, 708. 10.3389/fmicb.2020.00708 32362888 PMC7180184

[B40] Robinson-Dunn B. (2002). The Microbiology Laboratory’s Role in Response to Bioterrorism. Arch. Pathol. Lab. Med. 126 (3), 291–294. 10.1043/0003-9985(2002)126<0291:Tmlsri>2.0.Co;2 11860302

[B41] Shi L. Chowdhury S. M. Smallwood H. S. Yoon H. Mottaz-Brewer H. M. Norbeck A. D.. (2009). Proteomic Investigation of the Time Course Responses of RAW 264.7 Macrophages to Infection With *Salmonella Enterica* . Infect. Immun. 77 (8), 3227–3233. 10.1128/iai.00063-09 19528222 PMC2715674

[B42] Stanley N. R. Findlay K. Berks B. C. Palmer T. (2001). Escherichia Coli Strains Blocked in Tat-Dependent Protein Export Exhibit Pleiotropic Defects in the Cell Envelope. J. Bacteriol. 183 (1), 139–144. 10.1128/jb.183.1.139-144.2001 11114910 PMC94859

[B43] Wang F. Hu S. Liu W. Qiao Z. Gao Y. Bu Z. (2011). Deep-Sequencing Analysis of the Mouse Transcriptome Response to Infection With *Brucella Melitensis* Strains of Differing Virulence. PloS One 6 (12), e28485. 10.1371/journal.pone.0028485 22216095 PMC3247208

[B44] Wang Y. Li Y. Li H. Song H. Zhai N. Lou L.. (2017). *Brucella* Dysregulates Monocytes and Inhibits Macrophage Polarization Through LC3-Dependent Autophagy. Front. Immunol. 8, 691. 10.3389/fimmu.2017.00691 28659924 PMC5467008

[B45] Weatherspoon-Griffin N. Zhao G. Kong W. Kong Y. Morigen Andrews-Polymenis H.. (2011). The Cpxr/Cpxa Two-Component System Up-Regulates Two Tat-Dependent Peptidoglycan Amidases to Confer Bacterial Resistance to Antimicrobial Peptide. J. Biol. Chem. 286 (7), 5529–5539. 10.1074/jbc.M110.200352 21149452 PMC3037666

[B46] Yan X. Hu S. Yang Y. Xu D. Li H. Liu W.. (2020). The Twin-Arginine Translocation System is Important for Stress Resistance and Virulence of *Brucella Melitensis* . Infect. Immun. 88 (11). 10.1128/iai.00389-20 PMC7573438 32778612

[B47] Yoneyama M. Kikuchi M. Matsumoto K. Imaizumi T. Miyagishi M. Taira K.. (2005). Shared and Unique Functions of the Dexd/H-Box Helicases RIG-I, MDA5, and LGP2 in Antiviral Innate Immunity. J. Immunol. 175 (5), 2851–2858. 10.4049/jimmunol.175.5.2851 16116171

[B48] Zhang J. Li Q. Liu J. Lu Y. Wang Y. Wang Y. (2020). Astaxanthin Overproduction and Proteomic Analysis of *Phaffia Rhodozyma* Under the Oxidative Stress Induced by Tio_2_ . Bioresour. Technol. 311, 123525. 10.1016/j.biortech.2020.123525 32447228

