# Supplementary material for: Proteomics Investigation of the Time Course Responses of RAW264.7 Macrophages to Infections With the Wild-Type and Twin-Arginine Translocation Mutant Strains of Brucella melitensis
Source: Front Cell Infect Microbiol. 2021 Jun 14;11:679571. doi: 10.3389/fcimb.2021.679571 (PMC8238042; doi:10.3389/fcimb.2021.679571)
Supplement: Supplementary file 1 [file DataSheet_1.docx]

**Table S1 Strians and primers used in this study**

| Strains and Primers | Description | | Source or reference | |
| --- | --- | --- | --- | --- |
| Strains | |  | |  |
| *B.melitensis* M28 | | Parental strain; smooth phenotype | | Our lab stock |
| M28△*tatA* | | Deletion strain for *tatA* gene, km^r^ | | (Yan et al., 2020) |
| M28△*tatA* pBBR*tatA* | | △*tatA* strain carrying the plasmid pBBR-*tatA*, Amp^r^ | | (Yan et al., 2020) |
| qPCR-actin-F | | GGCTGTATTCCCCTCCATCG | | This study |
| qPCR-actin-R | | CCAGTTGGTAACAATGCCATGT | |  |
| qPCR-TNFα-F | | TTGTCTACTCCCAGGTTCTCT | | This study |
| qPCR-TNFα-R | | GAGGTTGACTTTCTCCTGGTATG | |  |
| qPCR-IL6-F | | TTTCCTCTGGTCTTCTGGAGTA | | This study |
| qPCR-IL6-R | | CTCTGAAGGACTCTGGCTTTG | |  |
| qPCR-iNOS-F | | CAGCTGGGCTGTACAAACCTT | | This study |
| qPCR-iNOS-R | | CATTGGAAGTGAAGCGTTTCG | |  |


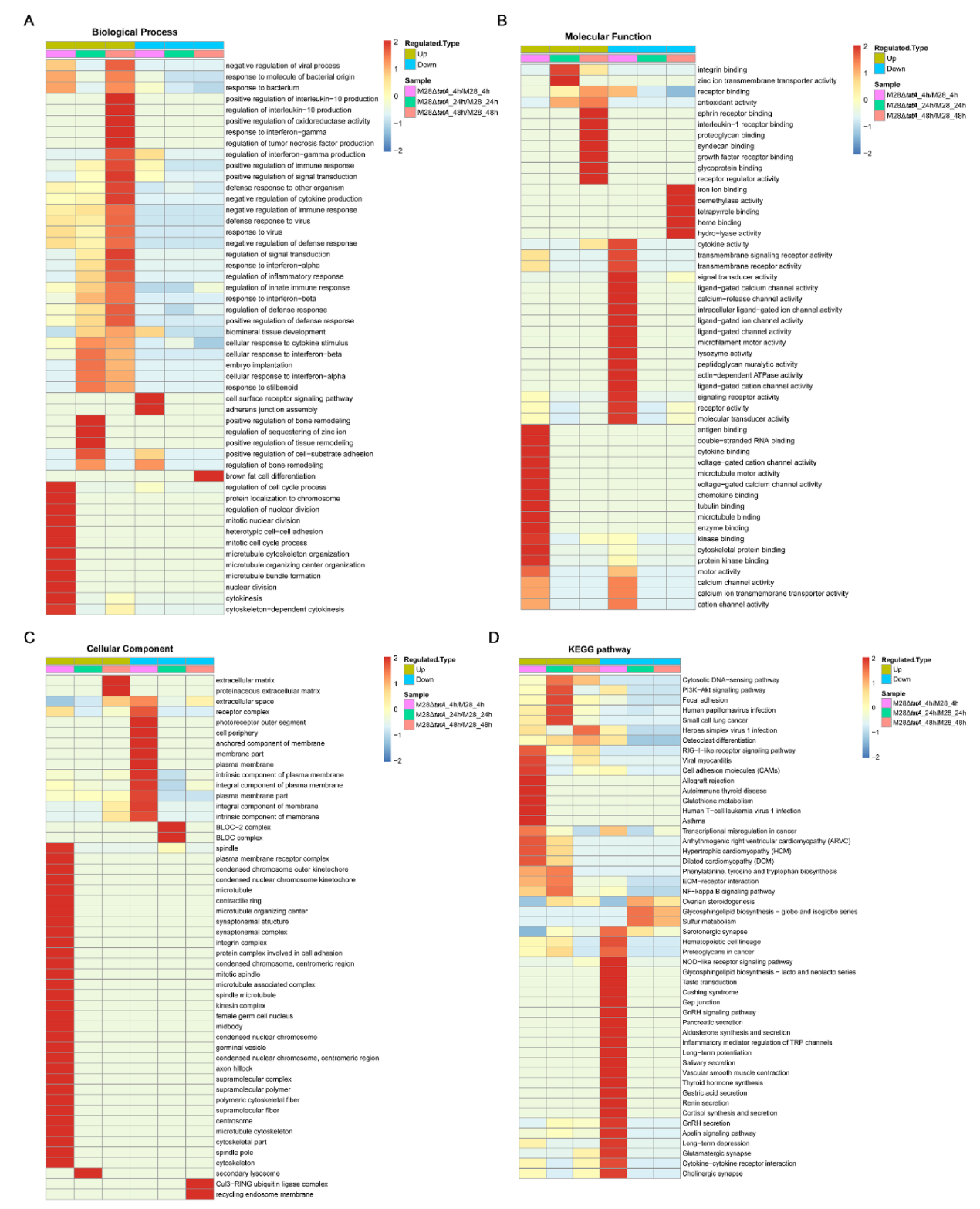


**Figure S1 Functional enrichment analysis of DEPs in RAW264.7 cells after infection with M28 and M28Δ*tatA.*** A-C, Cluster analysis heat map based on GO enrichment. D, Cluster analysis heat map based on KEGG enrichment. The horizontal direction represents the enrichment test results of the different parts, and the vertical direction is the description of the differential expression enrichment-related functions and KEGG pathways. Red indicates a strong degree of enrichment (the deeper the red, the stronger the enrichment), and blue indicates a weaker enrichment (the lighter the blue, the weaker the enrichment).


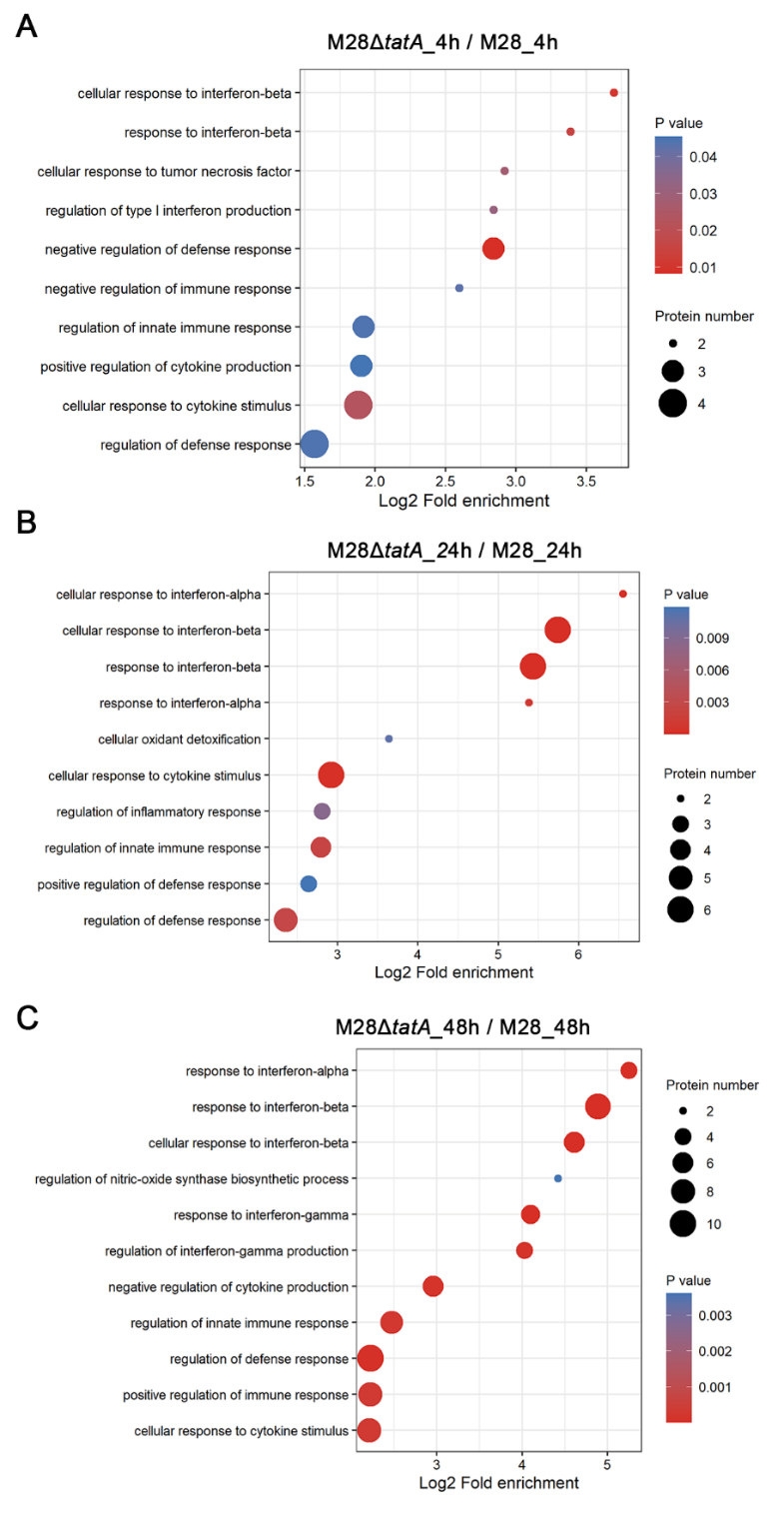


**Figure S2** **Biological process enrichment analysis of upregulated DEPs involved in the immune response in RAW264.7 cells after infection with M28 and M28Δ*tatA* 4 hpi (A), 24 hpi (B) and 48 hpi (C).** All DEPs shown were all significantly upregulated proteins compared to their counterparts in the M28Δ*tatA*/M28 group. The *x*-axis represents the log2-transformed fold enrichment, and the *y*-axis shows the description of the biological process enrichment with immune response proteins (top 10). Upregulated DEPs were subjected to the enrichment analysis based on Fisher’s exact test, and functional categories with p < 0.05 were considered significant. The area of the circle represents the number of differential proteins, and the color of the circle represents the p value obtained by Fisher’s exact test.

**
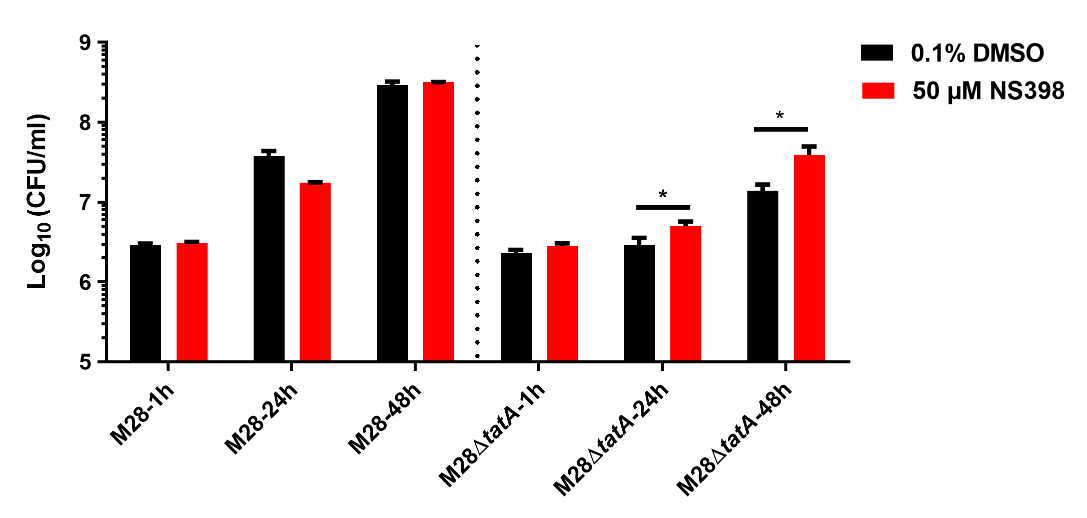
**

**Figure S3 Inhibiton of COX-2 increases the intracellular survival of Tat mutant *Brucella*.** NS398 treatment increased Tat mutant *Brucella* intracellular survival in RAW264.7 cells at 24h and 48h post infection; the data are shown as the mean ± standard error of the mean. Student’s *t* test was used to evaluated significant differences between the M28Δ*tatA* mutant and the wild type. * p＜0.05.

**References**

Yan X, Hu S, Yang Y, et al. (2020). The twin-arginine translocation system is important for stress resistance and virulence of *Brucella melitensis*. Infect Immun doi: 10.1128/IAI.00389-20.
